# Humans’ Relationship to Flowers as an Example of the Multiple Components of Embodied Aesthetics

**DOI:** 10.3390/bs8030032

**Published:** 2018-03-01

**Authors:** Ephrat Huss, Kfir Bar Yosef, Michele Zaccai

**Affiliations:** 1 Spitzer Department of Social Work, Ben Gurion University of the Negev, Beersheva 84105, Israel; kfirba@post.bgu.ac.il; 2 Department of Life Sciences, Ben Gurion University of the Negev, Beersheva 84105, Israel; mzaccai@bgu.ac.il

**Keywords:** arts-based research, embodied aesthetic experiences, arts in social work

## Abstract

This paper phenomenologically and qualitatively explores the relationship between humans and flowers as a relationship that throws light on the synergetic dynamics of embodied aesthetics. Its methods include qualitative description and thematic analyses of preferred flower types, as well as concept maps of the general term ‘flower’ by 120 students in Israel. The results revealed the interactive perceptual-compositional elements, as well as embodied, relational, and socially embedded elements of the aesthetic pleasure associated with flowers. Implications of this case study are generalized to understand the multiple and interactive components of embodied aesthetic experiences as a deep source of pleasure through interactive stimulation by and connection to the natural world.

## 1. Introduction

The relationship between humans and flowers is special. Humans have always been strangely attracted to flowers even when they provide no physical sustenance and when resources are low. Humans have also put embodied and physical effort into growing flowers for their aesthetic qualities. Stone drawings of flowers were found in ancient Egyptian graves 120,000 years ago, were celebrated in festivals in Roman times, and, in China, were created in silk 2000 years ago [1].

As stated, this attraction is not on a survival level, as while flowers can provide some basic medicinal uses and serve as a sign of the fertility of the land, the main motivation for growing flowers seems to be aesthetic. However, this goes beyond perceptual levels, as flowers are a multi-sensory experience that includes smell, texture, and color (and an embodied experience, in that one has to actively search for flowers with his/her body, to tend them, and to bend over them to smell them). The experience is also relational in that flowers are dependent on man’s care; they have to be handled gently, watered, and nurtured [2]. On all of these levels, the human interaction and the relationship with flowers seems to be an interesting example of embodied aesthetics. This perspective of the relationship between humans and flowers, and thus the incentive to grow them, is not well explored in the literature.

Indeed, embodied phenomenology assumes that our live bodies interacting with the environment are at the basis of our phenomenological experience of the world. Flowers are an apt example of being aware of the environment around us and of how we engage with the world through skilled interaction through our bodies—by using all of our senses. This happens through moving our bodies within space rather than only by contemplating the environment. Flowers, as described above, demand us to get close to smell them, to move towards them to find them in nature, to water them, to pick them, and to carry them in our hands. All of this embodied interaction makes them excellent examples and receptors for the experience of embodied aesthetics. Tending to and enjoying flowers thus enables us to interact in a skilled fashion with the environment and to engage with the world [3,4,5]. This behavior clearly creates positive emotions, as there is no survival-level incentive to engage with flowers. An exploration of this basic relationship to flowers may help us to understand how embodied aesthetics ‘works’ to enhance positive emotions in an ancient and enduring context of growing flowers [6].

As stated, aesthetic and embodied experiences include within them overt perceptual processes, but also, additional components that will be outlined below. Firstly, from an evolutionary perspective, the flower as a species uses, among others, the strategy of activating humans to grow and to propagate it, just as it activates insects with pollen. The plant thus uses its aesthetic characteristics to attract humans [7]. In turn, for humans, flowers may evoke positive emotions, because they can help predict food-growing possibilities and/or may be used as medicines. They essentially show where man can live healthily. In addition, colors of flowers may be helpful in finding ripe fruit against a green background [8,9]. However, as stated above, when man grows ornamental flowers, this evolutionary motivation is lessened. People have always actively invested resources in growing ornamental flowers since ancient times. 

Secondly, flowers have a strong visual component. Vision is a multimodal process that entails the activation, not only of the visual areas of the brain, but also of sensory-motor, viscera-motor, and affective cerebral circuits. On this level, flowers activate multiple parts of the brain creating a stimulating, perceptual experience [3]. The repeated, compositional elements of flowers such as color, shape, and pattern that are repeated within the petal arrangement and within a group of similar flowers growing in proximity are helpful in providing the right amount of familiarity and innovation to calm but also to activate the brain. Thus, flowers help us to actively organize perceptual experience [10,11]. This visual stimulation, together with the ease of recognition and the familiarity engendered by symmetry in flower shapes, may stimulate the brain and be associated with improved mood due to a feeling of being able to make sense of the world [12,13,14,15]. This combined element of familiarity and surprise is a basic component of aesthetic experience that is able to move us emotionally, activating both sadness and happiness. 

Thirdly, on a sensory level, this aesthetic perceptual pleasure in flowers goes beyond vision to include smell, movement, and sensory stimuli. Flowers evoke a multisensory experience, as shown in watching flowers sway in the wind and their use in perfume [16,17].

Fourthly, these sensory stimuli also stimulate autobiographical memory, creating a web of positive associations around flowers through former experiences with them or components of them [15,18]. For example, color, smell, and shape connect to autobiographic memories and stimulate the recall and accessibility of long term memory [12,18,19,20]. Autobiographical memory is important for this understanding of flowers, as flowers triggers a sensory aesthetic experience that connects to previous autobiographical memories of interactions with them. These cultural levels cut through the strong sensory association with previous positive experiences as learned associations of flowers. This may be the reason flowers are connected to positive social events, such as romance and celebrations within the context of specific cultural constructions of meanings [21]. 

Fifthly, from this, flowers as embodied aesthetics become socially embedded. They enhance pleasant stimulation of the brain that is then connected to positive social experiences. This can be a central incentive of humans to culture flowers [1]. In one study, positive emotions were maintained for three days after receiving flowers and made people likely to smile and create more social contact when given flowers [22,23]. Flowers are thus connected to a positive, emotional environment for optimal psychological—and from this, physical—health. We tend flowers as we tend loved ones. The aesthetic experience becomes socially embedded and relational. People become happier when given a large bunch of flowers by a loved one as opposed to when given money. The embodied sensory level of flowers seems to be connected to the positive element of relationship, from romantic and sexual, as well as to general positive, social interactions [24]. From this, we can surmise that the aesthetic experience of flowers leads to the embodied and socially embedded experience of feeling connected positively to the world.

Finally, on the level of the connection between embodied aesthetics and resilience, resilience can be enhanced through focusing on positive thoughts and emotions. This may be a hint as to the real motivation behind cultivating flowers—to create a positive experience of the world. Indeed, art therapy and nature therapy utilize visual elements such as mandalas and positive images from nature to create a ‘safe place’ to regulate emotions. These embodied aesthetic experiences have healing qualities in themselves in that they re-connect to positive, social experiences through stimulating the mind, senses, and body to interact positively with others. We saw that aesthetic experiences enable the regulation of physiological and emotional over- and under-excitation of the organism. This is expressed in theories of creative processes such as art-making or observing, enabling a mind-set of ‘flow’ and deep concentration, as well as regulated communication with others. Art-making and observing has been defined as an integrative activity that integrates left and right brain functions, and, as such, creates new neurological pathways between emotional and cognitive areas of the brain, enabling flexibility of thought, as opposed to the rigid, repetitive, or fragmented thinking when under stress or after trauma [25,26,27,28]. These ideas are transferred to nature in nature therapy and horticulture therapy, both utilizing the outside to create this embodied positive interaction with the environment through immersion in the outside and in growing plants. However, the literature on these areas tends to focus more on the psychosocial and relational corrective value of these activities when undertaken in a therapeutic environment, rather than focusing on the inherent characteristics of embodied aesthetics as a socially embedded phenomenon [28].

## 2. Materials and Methods

Our aim in this study is to further deepen our understanding of the specific interaction between humans and flowers as embodying a positive experience of its own. In our first paper [29], we compared mandalas to flowers in terms of the types of reactions they evoked. We found that, while mandalas were described by the participants as arousing more perceptual interest, flowers created more feelings of happiness. The themes in this former study pointed to the embodied nature of the experience of flowers, even when presented only as pictures. Based on this, we wished to analyze our data through the theoretical lens of embodied aesthetics. Does embodied aesthetic theory explain the flowers’ inherent characteristics that are at the base of this positive experience? What interaction is there between physical, emotional, cultural, and embodied elements that are ‘invisible’ but underneath man’s aesthetic pleasure in flowers? We utilized qualitative research, because we wanted a preliminary understanding of the phenomenological experience of flowers, rather than to test specific hypotheses [30,31]. The research, thus, used a qualitative methodology to ask how and in what ways the relationship between people and flowers can be conceptualized through theories of embodied aesthetics. It focused on the phenomenology of how people self-define their experience of flowers. These preliminary and open questions are suited to qualitative methodology. Further research can create hypotheses from this data. 

The current study used two stages of research:

- Stage 1: A written explanation of choice of preferred flower from between four different flowers.
- Stage 2: Use of a concept-map of the meanings of the word ‘flower.’

### 2.1. Field of Research

Participants consisted of 60 men and 60 women, all undergraduate students at the Ben-Gurion University of the Negev. Some of them were students at the Department of Social Work, and the remaining participants were recruited by these students who were part of a research seminar. 

Stage I: Preferred Choice of Flower from among Four Different Flowers. We wished to understand the meaning and reasons attributed to first choice flowers, and so we asked the participants to choose between four different flowers and to explain this choice in an unstructured sentence. We chose two radially symmetrical-shaped flowers, one with relatively few petals (*Gerbera*) and one with many layered whorls of petals (*Ranunculus*). We also chose two bi-laterally symmetrical and elongated shapes, the Calla lily and the *Anthurium*. These flowers are cultivated for ornamental purposes and widely found in flower shops, and so are familiar to the participants. The 120 participants were then asked to choose from among the four black and white pictures of flowers and to provide unstructured written explanations for their first choice. As for analytical strategy, we counted the different preferences in flowers and, interestingly, found that the four flower types were equally distributed in terms of first choice [29]. 

Stage 2: Concept Sheet on ‘Flower’. From the findings of Stage 1, we wanted to further explore the central finding of an overriding ‘flower’ quality that transcended the flowers in light of the equal distribution among the four flowers, rather than focus on the differences between flowers. We chose a concept map in order to understand the general concept of a generic flower for people and asked 40 participants to write a concept map [30] that included writing associations of the word ‘flower’ for five minutes without stopping. 

Within this stage, we asked this set of students to

(1) Write any words that come up when the word ‘flower’ is said for two minutes,
(2) Fill in half a page concerning the emotions connected to a flower, and
(3) Fill in half a page concerning memories that include a flower.

After this, there was an open group discussion about what participants wrote that was recorded and that elaborated upon their concept map. The aim of this task was to deepen our understanding of generalized concepts of what is a flower. For this stage, our data included 40 concept maps of the participants. We thematically analyzed and categorized the words from the concept map, the themes in the structured writing, and the themes from the discussion. These associations were divided into central themes. The main finding was that flowers were associated with the words ‘pretty’ and ‘make me happy’ [29]. 

### 2.2. Ethical Considerations

The flower interviews, concept sheets, and questionnaires were one of the participant’s non-mandatory assignments within a research seminar. Participants were not named, and personal data was not gathered or used. The participants signed consent forms in order to fill out the questionnaire. This paper does not have ethical problems as it discusses the neutral area of flowers and does not use any experimental methods. It was also implemented in a research class in which the students were given credit for participating in research. We received signed agreement to participate in the assignment, and we received ethical clearance from the Ben Gurion University departmental ethics committee.

### 2.3. Validity and Reliability

Because we approached the research with different qualitative tools, such as semi-structured free writing as part of our comparison of flowers questionnaire, counting, and concept sheets, we created a triangulation or validation of the data [30]. We utilized peer evaluation of different experts, such as a biology researcher specializing in flowers, an arts-based researcher specializing in qualitative research and visual elements, and a social scientist researcher specializing in psychological and social theories. This peer analysis also created reliability. As stated, this research section was part of a larger study that included additional data analyses (see [29]). 

## 3. Results

**Figure 1:**
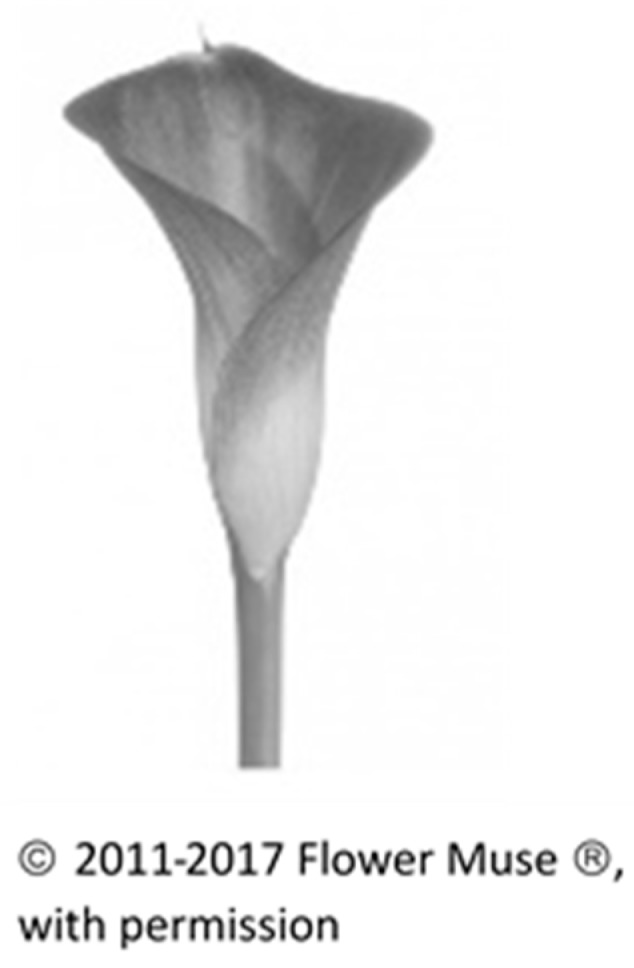
Calla.

Our first set of themes compared the four different flowers. We saw that different characteristics were attributed to each flower-type. These personified the flower as a specific ‘personality.’ The first flower was defined as elegant and mysterious (*Calla*, an elongated lily shape) (see Figure 1):

Following are the comments concerning the Calla:

“You want to bend over it and explore it.”

“Shy, closed, interesting.”

**Figure 2:**
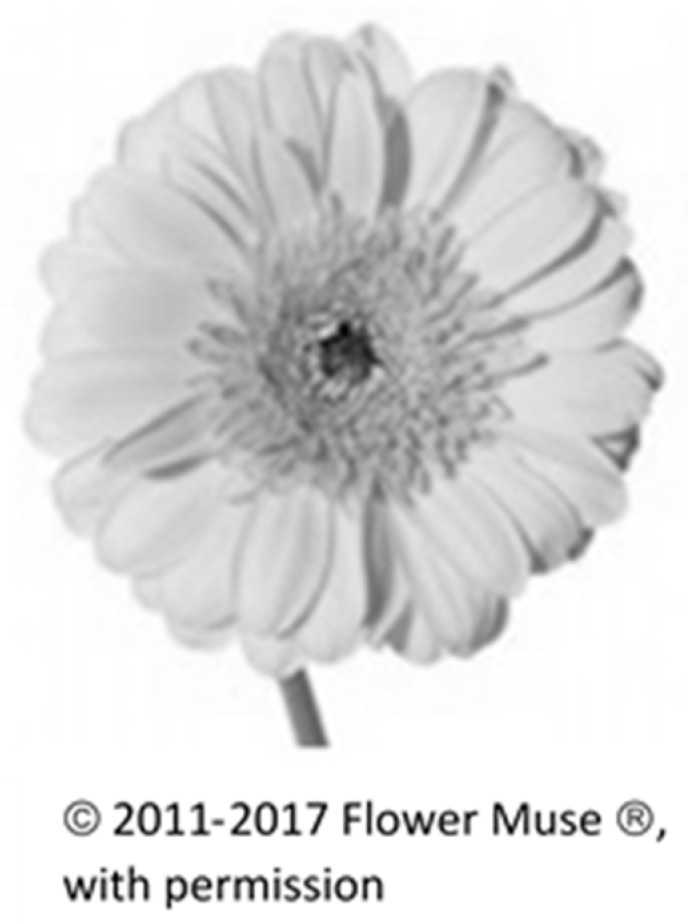
Gerbera.

The second was defined as simple and wholesome (*Gerbera*, a simple daisy flower) (see Figure 2):

Following are the comments on the Gerbera:

“It gives me security.”

“Something pure and simple and optimistic.”

**Figure 3:**
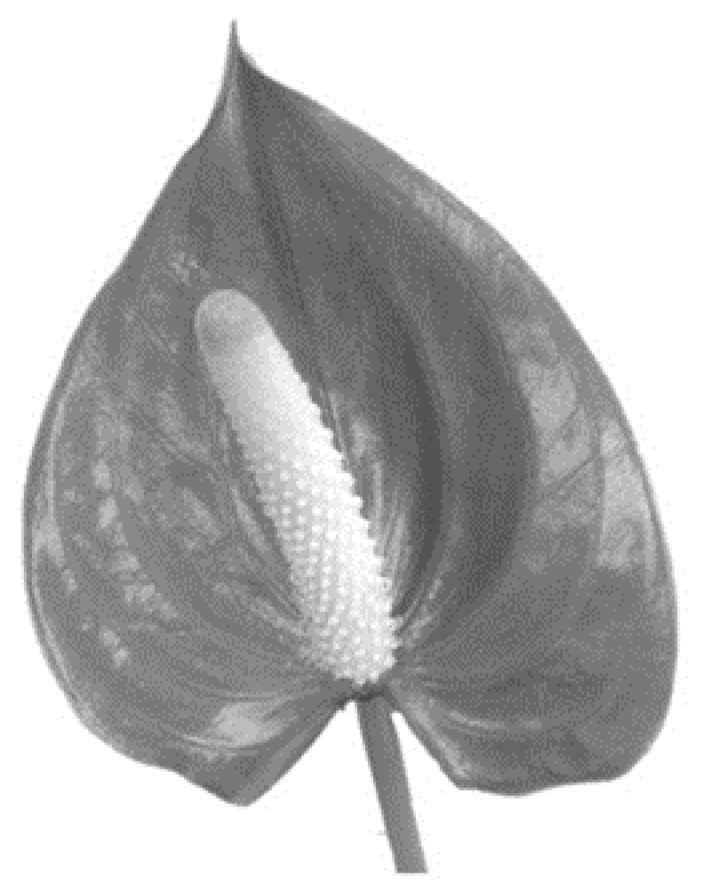
Athurium

The third was defined as authentic (*Athurium*, a green, leaf-like flower) (see Figure 3):

Following are the comments for this flower:

“It is connected to home and to family.”

“It is modest, not fancy, and the most real.”

**Figure 4:**
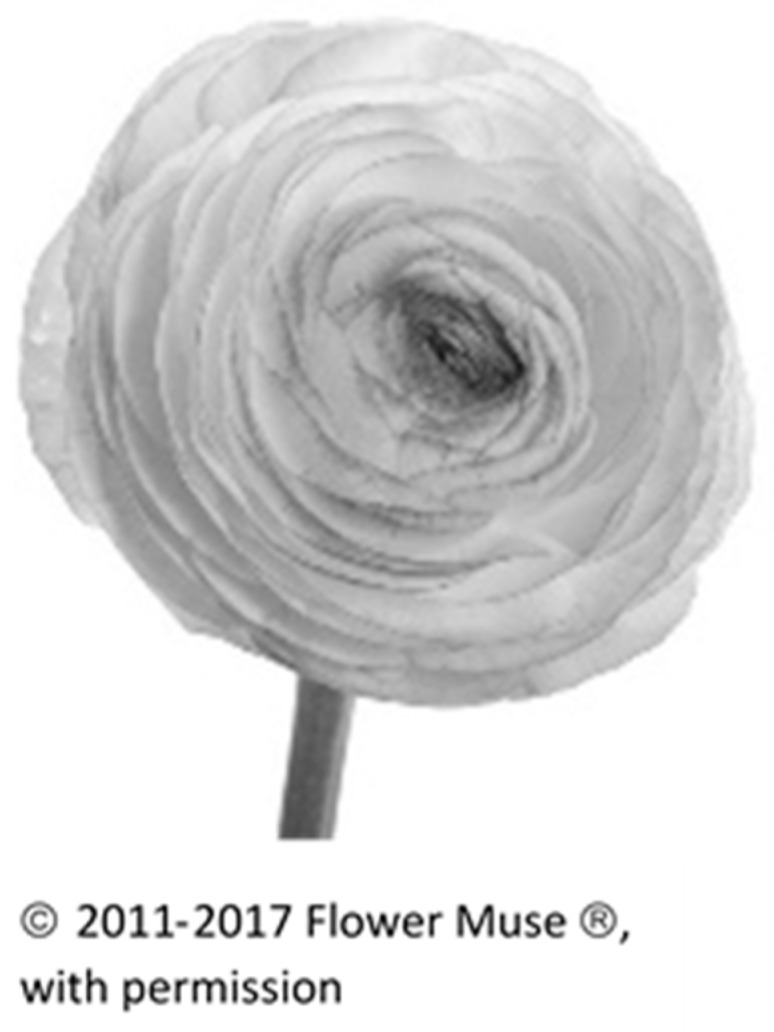
Rananculus.

The final flower was defined as sensuous and feminine (*Ranunculus*, a flower with strong colors and many petals) (see Figure 4):

Following are the comments for this flower:

“I chose it because I can imagine it has a good smell.”

“I am drawn to its softness. It looks so nice to touch.”

## 4. Discussion and Conclusions

We see above that the specific, physical characteristics of the flower are engaged with and create a ‘personality’ for each flower. This is considered as embodied, because it goes beyond perceptual projections to include sensory elements such as smell, shape, and color. The flower becomes a full ‘body’ with which to interact. This is further elucidated by the findings of our previous paper (see [29]), in which we compared flowers to mandalas and found that, while mandalas were defined as perceptually more interesting, flowers made people happy and calm. We can explain this difference through the relational and embodied perspective outlined in this current paper, as the embodied and relational nature of the flowers enables one to project personalities on them and then to interact with them as a ‘body in space.’ This was shown above to be both a personal body as well as a cultural, collective entity. We saw that some flowers received a set of positive and collective Israeli values such as “simplicity and directness—the importance of family and the importance of connection to the land of Israel” (see findings). These are also embodied in that they focus on a strong collective relationship and on exploring the land with one’s feet, meeting flowers in nature as an embodied endeavor, rather than an abstract perceptual, cultural, or cognitive symbolic element as in the quotes presented throughout this section.

Interestingly, these collective values connected to the collective values of family, country, and religion, as opposed to focusing on the romantic values of flowers, as was shown to be central in European and Western cultures [24]. This finding corresponds to Derridas’ [32] definition of some concepts as ‘signified’—that is, holding dense cultural meaning, in this case, both personal and collective. These themes included flowers characterized by human qualities such as ‘gentleness’, flowers characterized by group characteristics such as ‘family values’, flowers that symbolize a relationship such as reminding one of a parent because ‘*I bring her flowers on Friday night*’, and flowers as making a regional event special. These cultural projections were shown, however, to be in a synergetic embodied relationship with the perceptual elements of the flower, such as through color and shape. 

From this, it is possible to see that the perceptual, compositional elements of flowers are connected to the abstract characteristics that correspond to them, and this helps to give meaning to and actively organize perceptual as well as cultural experience [10,11,12,13,14,15]:

“Its shape is simple, open, and direct; it is not hiding anything, and there are not hidden layers of petals. It is round, and all parts are equal.”

However, our point is that the flower goes beyond a perceptual and cultural interaction to a more embodied and relational type of experience. The most central description of the experience of flowers was that flowers make people ‘happy.’ While there is, as shown above, aesthetic pleasure in connecting between perceptual and cultural integration and between familiarity and innovation, the concept map defined the overarching characteristic of flowers as making people ‘happy.’ This is because they symbolize but also enact the embodied relationship between people (through looking after the flower, bending over it, giving it water, and carrying them to meaningful relational, personal, and cultural events such as to a family meal or a funeral):

“Flowers remind me of childhood, of trips, of being together, of searching for the first spring flowers, of national flowers that can’t be picked.”

“I was stressed but when my boyfriend bought flowers, I knew it would be a special evening.”

Our findings, when read through theories of embodiment, point to the fact that the relationship between people and flowers goes beyond the synergetic connection between the visual characteristics and the social meanings of the flower, to the relational, embodied element of person-flower connection that is embodied in physical characteristics. These are what enable to create the ‘floweriness’ of the flower as defined as able to ‘make me happy.’

Through observing the relationship between person and flower from an embodied perspective, we learned that flowers demand us to become engaged with the flower, and thus the world, on the embodied level, helping to bridge moments of embodied separation from people [33]. Flowers demand the interaction and engagement of the bodies of people to tend them, to move towards them, to bend over and smell them, to pick them, and to look after them. This corresponds to definitions of embodied aesthetics that occur through moving our bodies within space, rather than only through contemplating the environment. Flowers enable us to interact in a skilled fashion with the environment [3,4,5].

Flowers were mentioned as being connected to meeting loved ones, to separating from them, and to making social gatherings special. This is embodied, as well as symbolic, because flowers have sensory elements such as softness and smell—just as Winnicott’s [34] transitional objects have:

“A flower can be held. It can be smelt. It is gentle, and you need to look after it.”

“It has a lot of petals in layers, and it makes one want to touch it.”

“It has a good smell.”

“It is delicate; it needs to be handled carefully.”

The themes of sensory engagement with a flower such as smell, softness, and color, as well as the relational elements of caring for the flowers, i.e., that it needs to be looked after, watered, sheltered from wind, etc., create a physical embodied relationship that folds within it conceptual, perceptual, cultural, and other elements. But what makes people ‘happy’ is the combination of these in an embodied context.

One of this study’s limitations was that we used images of flowers rather than real flowers. However, the aim of the research was not to explore the embodied element of human-flower relationships; rather, the theme of embodied aesthetics emerged as an apt theory to explain the findings, even in a ‘non-embodied’ framework, that emerged from the phenomenological narratives of the participants. This further strengthens the validity of this chosen direction. As is known, within qualitative grounded research the findings can, and hopefully do, lead to new theoretical explorations that expand the original direction [31]. 

Another limitation could be the explorative preliminary nature of this study, which started by comparing flowers and moved on to a concept map of the meaning of the term ‘flower.’ However, the two stages of data gathering validated each other with recurring themes. Future research can expand and be replicated in other countries in terms of culturally embodied meanings attributed to flowers and through utilizing embodied methods. 

The wider contribution of this study is that it provided a qualitative phenomenological exploration of the meaning of flowers that pointed not only to the components of embodied relationships but also to socially embedded aesthetics. We saw the synergetic connection between perception, autobiographic memory, senses, and physical movement in our relationship with flowers, but most importantly, we saw that the pleasure effect of flowers was connected to the physically interactive and relational elements of the experience. This means that the perceptual elements of the flower are used as a projective element for a specific personal and cultural personality; however, the point is that what caused the flowers to be defined as ‘happy’ was the embodied and relational experience of the flower. This point is strengthened by and helps to explain the finding in our first article on flowers that compared flowers to mandalas and found that while flower shaped mandalas were defined as interesting, flowers were defined as ‘calming’ and ‘making one happy.’ Through using an embodied theory, we see how the happiness element emerges from the holistic, sensory, embodied, and relational elements of the human-flower interaction as compared to the perceptual elements of a mandala [29].

Implications of this paper can also be relevant for positive psychology. They suggest that embodied relational experiences cause people to feel ‘happy’ [5,25,35]. These findings can then be integrated into positive psychology’s methodologies, connecting them to the above literature on the ecological conception of humans as a social and communicative species that also communicates with their environment, looking after and tending decorative flowers in return for their embodied and socially embedded elements that enhance one’s experience of self in the world. This has implications as a theoretical base for including nature, movement, gardens, cooking, and other sensory, relational, and holistic actions within positive psychology methods, rather than staying within the conceptual or aesthetic framework alone.
